# Leukocyte infiltration patterns and structural changes in severe asthmatics with variable degree of clinical control

**DOI:** 10.1186/2045-7022-5-S2-O7

**Published:** 2015-03-23

**Authors:** Bergqvist Anders, Cecilia Andersson, Hans-Jürgen Hoffmann, Michiko Mori, Medya Shikhagaie, Inge Kortekaas Krohn, Ronald Dahl, Leif Bjermer, Jonas Erjefält

**Affiliations:** 1 Lund university, Department of Respiratory Medicine and Allergology, Lund, Sweden; 2 Aarhus university, Department of Pulmonary Medicine, Aarhus, Denmark; 3 Lund university, Department of Experimental Medicine, Lund, Sweden; 4 Aarhus university, Department of Respiratory Disease, Aarhus, Denmark; 5 Lund university hospital, Department of Respiratory Medicine and Allergology, Lund, Sweden

## Background

A subset of patients with severe asthma (SA) remains persistently symptomatic despite treatment with inhaled and oral administration of corticosteroids. The aim of present study was to map the pattern of infiltrating leukocytes and study structural parameters in the bronchi of patients with this type of asthma.

## Method

Bronchial biopsies were obtained from 25 SA patients that were classified as either stable (n=15) or symptomatic (n=10). Biopsies from 8 healthy subjects served as control material. Tissue sections were stained in an automated immunohistochemistry robot and subsequently analysed with computerized image analysis.

## Results

In the comparison between stable SA and symptomatic SA, stable SA was associated with higher levels of eosinophils (p<0.05), T-helper type-2 cells (p<0.05) and macrophages (p<0.01). The remaining cell types analysed; T-helper cells, T-cytotoxic cells, B-cells, natural killer cells, neutrophils, basophils and mast cells tended to be lower in symptomatic SA compared with stable SA, although no statistical significance was found. Comparing the two SA groups with healthy controls, stable SA was associated with significantly increased levels of several leukocyte populations whereas symptomatic SA was not. Interestingly, both SA groups were characterized by a significant epithelial metaplasia as compared with healthy control subjects. Reticular basement membrane thickness and airway smooth muscle mass was similar between all three groups, although some patients in the SA groups had high levels. Within the SA group (n=25), a significant correlation was found between FEV1/FVC and the expression of myosin light chain kinase in the airway smooth muscle mass (r=0.66 and p<0.001). The two SA groups were matched in terms of gender, age (median 51, p=1.0) years), BMI (median 28.4, p=0.3), FEV1/FVC (median 0.67, p=0.2), dose of oral corticosteroids (median 15 mg/d, p=0.8) and duration of disease (median 16 years, p=0.9).

## Conclusion

The present study indicates that, in contrast to stable SA, there are no signs of chronic cellular tissue inflammation in the bronchi of severe asthmatics with persistent symptoms. The finding of epithelial metaplasia indicates that intermittent external assaults may play an important role in these patients disease.

